# First report of *Thelazia callipaeda* infection in wild European rabbits (*Oryctolagus cuniculus*) in Portugal

**DOI:** 10.1186/s13071-016-1526-1

**Published:** 2016-05-10

**Authors:** Adelina Gama, Isabel Pires, Márcia Canado, Teresa Coutinho, Ana Patrícia Lopes, Maria Stefania Latrofa, Luís Cardoso, Filipe Dantas-Torres, Domenico Otranto

**Affiliations:** Department of Veterinary Sciences, Laboratory of Histology and Pathology, School of Agrarian and Veterinary Sciences, University of Trás-os-Montes e Alto Douro (UTAD), Vila Real, Portugal; Animal and Veterinary Research Centre (CECAV), UTAD, Vila Real, Portugal; Municipality of Vinhais, Vinhais, Portugal; Department of Veterinary Sciences, Laboratory of Parasitology, School of Agrarian and Veterinary Sciences, UTAD, Vila Real, Portugal; Department of Veterinary Medicine, University of Bari, Valenzano, Italy; Department of Immunology, Aggeu Magalhães Research Centre, Oswaldo Cruz Foundation, Recife, Pernambuco Brazil

**Keywords:** *Thelazia callipaeda*, Eye worm, *Oryctolagus cuniculus*, European rabbit, Portugal, Wild, Zoonosis, *cox* 1

## Abstract

**Background:**

*Thelazia callipaeda* is a zoonotic nematode that affects the eyes of domestic and wild animals, including dogs, cats and red foxes. This parasitic eye worm is transmitted by *Phortica variegata*, which is a zoophilic fruit fly spread in Europe. Two wild European rabbits (*Oryctolagus cuniculus*) found dead in north-eastern Portugal were submitted to necropsy.

**Results:**

Both animals presented gross lesions compatible with haemorrhagic viral disease. Eye examination revealed the presence of six worms (three in each animal, on both eyes). Out of the six nematodes, five females and one male were morphologically and molecularly identified as *T. callipaeda*.

**Conclusions:**

This is the first report of *T. callipaeda* in wild rabbits from Portugal, which reveals a new host for this parasite in southern Europe and emphasizes the importance of including thelaziosis in the differential diagnosis of ocular alterations in both animals and humans from areas where the eye worm is endemic.

## Background

*Thelazia callipaeda* (Spirurida, Thelaziidae), also known as the “Oriental eye worm”, is a zoonotic nematode first described in East Asia and in the eastern end of the former Soviet Union [1]. Originally recognized in Far Eastern countries, it is now acknowledged that the parasite is also widespread in western European countries [2]. Following its description in dogs, cats and foxes in Italy [3], *T. callipaeda* has been reported in either domestic or wild hosts from France [4, 5], Switzerland [6], Spain [7], Bosnia and Herzegovina, Croatia [8] and Romania [9].

*Thelazia callipaeda* is transmitted by *Phortica variegata* (Diptera, Drosophilidae, Steganinae), a drosophilid insect that feeds on lachrymal secretions of mammals. The eye worm is larviparous and transmitted exclusively by those secretophagous flies that, during daytime and after landing on the eyes, release the infective larvae on the host’s conjunctiva [10–12]. Due to the presence of this nematode in the conjunctival sac, infection may lead to ocular manifestations ranging from mild conjunctivitis, epiphora and ocular discharge to severe keratitis and corneal ulcers in animals and humans [13, 14].

Infection in wild carnivores, including red foxes (*Vulpes vulpes*), beech martens (*Martes foina*), wolves (*Canis lupus*) and wildcats (*Felis silvestris*), may play a role in the geographical distribution of this nematode, by maintaining and spreading the parasite into previously non-endemic countries and regions of Europe [15, 16].

In Portugal, cases of infection have been described in domestic animals (i.e. dogs and cats) and red foxes from inland areas of the north and central regions of the country [17–22]. To the best of our knowledge, no human cases have been reported in Portugal, though they were reported in western Spain near the Portuguese-Spanish border [23]. In spite of the fact that leporids may be potential hosts for this parasite [15], reported cases of *T. callipaeda* infection in those vertebrate hosts in Europe are scarce, with just one reference of infection in brown hares (*Lepus europaeus*) [16]. Here we described the first cases of *T. callipaeda* infection in wild European rabbits (*Oryctolagus cuniculus*) in Portugal.

## Methods

Two wild rabbits (one female and one male) found dead at Vilar de Ossos, in the municipality of Vinhais, north-eastern Portugal (41°88′25.40″ N, -07°02′18.62″ W, and 41°86′14.06″ N, -07°01′96.44″ W), were sent frozen by the local authorities to the Laboratory of Histology and Pathology of UTAD, Vila Real, in order to have their cause of death determined.

Morphological identification of worms was done according to the keys proposed by Otranto et al. [24]. Briefly, *T. callipaeda* females have a vulva anterior to the oesophagus-intestinal junction, whereas males possess five pairs of postcloacal papillae. Collected worms were soaked in tap water for 24 h and then stored in 70 % ethanol.

Four nematodes (three females and one male) were subjected to specific PCR amplification of a portion (689 bp) of the cytochrome *c* oxidase subunit 1 (*cox*1) gene [25]. Amplicon sequences were determined in both directions (using the same primers individually as for the PCR) by visual inspection of the individual electropherograms. Sequences were aligned using the ClustalW program [26] and then compared with those available in public databases by BLAST analysis (http://blast.ncbi.nlm.nih.gov/Blast.cgi).

## Results

**Fig. 1:**
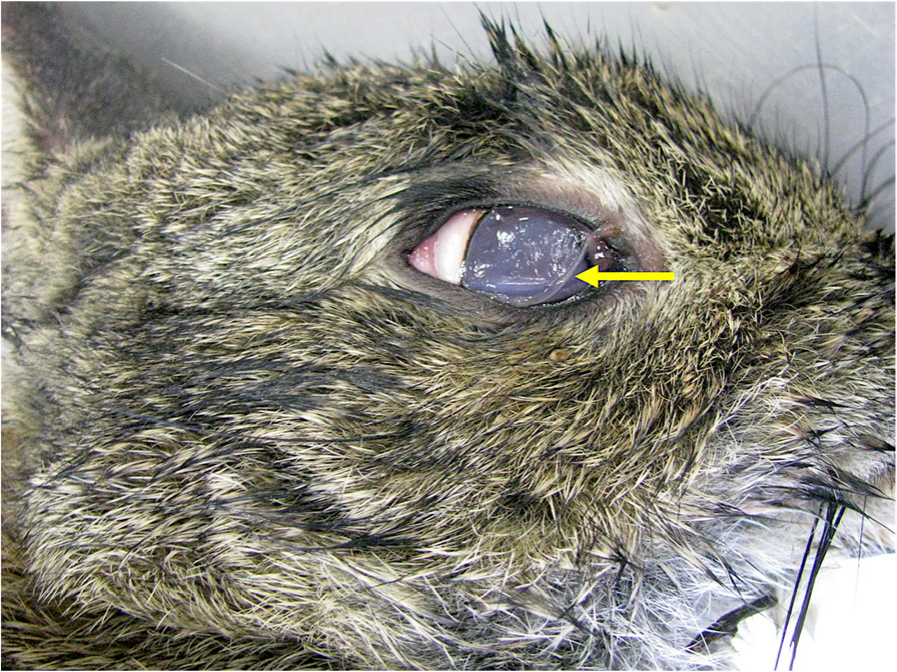
Eye of wild European rabbit. *Thelazia callipaeda* adult worm (*arrow*)

**Fig. 2:**
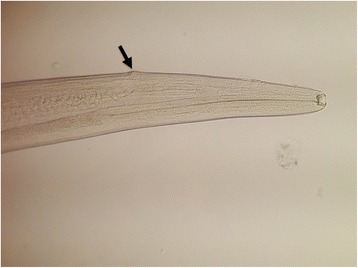
Adult female *Thelazia callipaeda*. Anterior part with vulva (*arrow*)

**Fig. 3:**
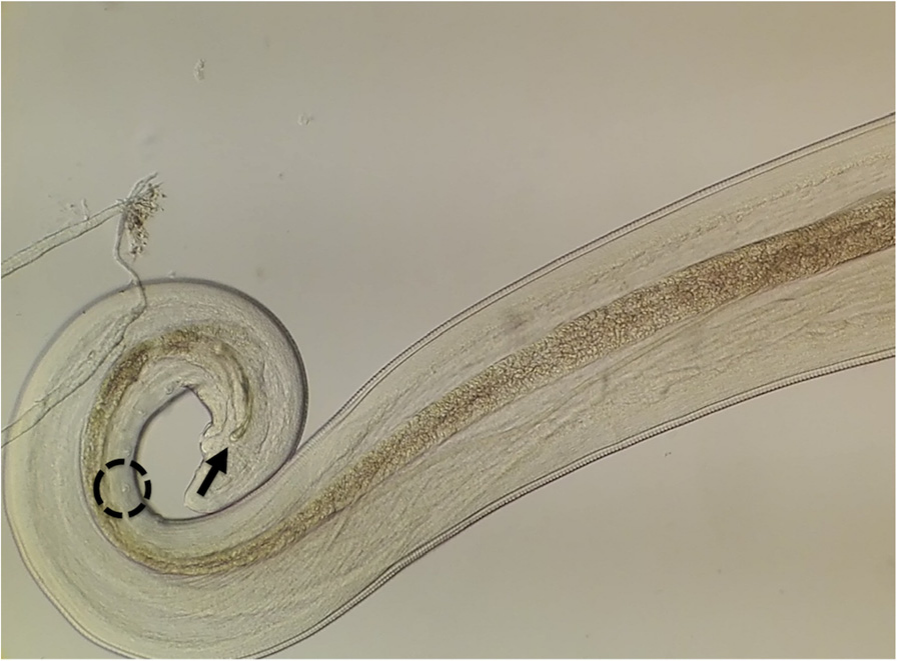
Adult male *Thelazia callipaeda*. Posterior part with postcloacal papillae (*dashed circle*) and spicule (*arrow*)

At necropsy, after thawing, both animals (with 1.2 kg of body weight for the female and 1.1 kg for the male) presented nose bloody discharge, splenomegaly and haemorrhagic lesions, mostly in lungs and trachea, compatible with haemorrhagic viral disease. In addition, eye examination revealed the presence of a total of six nematodes (three in each animal, on both eyes) (Fig. 1). Out of the six worms, five were morphologically identified as female (Fig. 2) and one as male (Fig. 3) *T. callipaeda*.

The morphological identification was confirmed by molecular analysis. A representative sequence (GenBank accession no. KX033489) obtained from all the nematodes examined was identical to *T. callipaeda* haplotype 1 (GenBank accession no. AM042549).

## Discussion

This is the first report of *T. callipaeda* infection in wild rabbits in Portugal. To our knowledge, only Skrjabin et al. [27] (cited by Otranto et al. [16]) described *Thelazia* infection in rabbits. Otranto et al. [16] investigated the occurrence of this infection in wildlife species from Italy and reported for the first time *T. callipaeda* in brown hares (*L. europaeus*), a species that belongs to the same family (Leporidae) of the European rabbit.

This report of *T. callipaeda* on the eyes of wild European rabbits indicates this species as a novel recognized host for this nematode in southern Europe. Thus, it confirms and amplifies the wide range of definitive hosts of *T. callipaeda*, as previously stated by other authors [16]. Although their population sizes are not accurately ascertainable at the regional and local levels (N. Fernandes, personal communication), wild rabbits are fairly common animals across the country in Portugal. In the near future, it will be important to investigate the prevalence of this infection in wildlife in Portugal at the population level, by more thoroughly assessing wild rabbits and red foxes [22], which have been found infected and could play a role in the dissemination of *T. callipaeda* in this country [15].

The infected wild rabbits described in this study were found in a geographical area which is environmentally comparable to other European areas where thelaziosis is currently considered as endemic, such as Spain [7], France [4] and Italy [16, 28]. Ocular *T. callipaeda* infection has also recently been described in dogs from the same municipality (Vinhais) and other contiguous municipalities (Chaves and Bragança) [19]. All these findings indicate that this region provides suitable habitats for the vector *P. variegata* [29]. However, further studies are necessary to investigate the ecology of *P. variegata* in northern Portugal.

Although no cases of human thelaziosis have so far been reported in Portugal, an increasing number of case reports in domestic and wild animals suggests the likely risk also for humans, especially in the north and central regions of Portugal, where several animal cases of infection have been described [17–22]. *Thelazia callipaeda* infection may cause lacrimation, epiphora, conjunctivitis, keratitis and even corneal ulcers [13], and should always be considered in the differential diagnosis of ocular alterations in both animal and human species, especially in endemic regions. The cases described in this report had no ocular alterations that could be associated with the presence of the eye worm; however, if and when present, potential ocular alterations may hamper the vision of the affected animals rendering them more vulnerable to predators.

Preventive measures to avoid eye worm transmission should be investigated in wild animals. With regard to canine species, the administration of systemic macrocyclic lactones (e.g. milbemycine oxime and moxidectin) may be recommended, whereas the use of slow-release insecticide collars do not protect against thelaziosis [30]. Finally, the European rabbit might constitute an experimental model for the study of potential therapeutic and preventive agents against *T. callipaeda*.

## Conclusions

The present findings reveal a new host for *T. callipaeda* in southern Europe and provide new clues to the epidemiology of *Thelazia* infection in wildlife species. Considering the growing number of cases described in domestic and wild animals, frequently in close contact with humans, thelaziosis should be included in the differential diagnosis of ocular diseases both in animals and humans.

## Ethical approval

All the procedures in this study were in accordance with the Portuguese legislation for the protection of animals (Decree-Law n° 113/2013).
